# Large left atrial thrombus resection in a patient in sinus rhythm without mitral valve disease: A case report

**DOI:** 10.1016/j.ijscr.2022.107000

**Published:** 2022-03-30

**Authors:** Hironobu Nishiori, Yuichi Hirano, Masayoshi Otsu, Hiroyuki Watanabe

**Affiliations:** Division of Cardiovascular Surgery, Narita Red Cross Hospital, 90-1, Ida-Cho, Narita City 286-8523, Chiba, Japan

**Keywords:** Left atrial thrombus, Myxoma, Atrial fibrillation, Mitral valve disease, Case report

## Abstract

**Introduction:**

Left atrial (LA) ball thrombi are often associated with atrial fibrillation (AF) and mitral valve disease (MVD). Differentiating between thrombi and LA tumors can be challenging.

**Presentation of a case:**

A 63-year-old man with a prior mesh insertion for abdominal incisional hernia was admitted with fever. He was diagnosed with an abdominal mesh-related infection requiring surgical debridement. Preoperative transthoracic echocardiography revealed a 39-mm smooth mass in the LA adherent to the atrial septum. The mass was suspected to be a cardiac tumor based on the morphology. The patient underwent mass resection. Pathophysiology revealed that the mass was a thrombus, necessitating anticoagulation therapy. No recurrence of thrombus formation was reported.

**Discussion:**

In this case, a plausible factor causing the thrombus formation is the chronic mesh. Since LA thrombi can become free-floating or grow rapidly, early surgical intervention is essential to prevent thrombotic events or sudden death.

**Conclusion:**

An LA thrombus should be included in the differential diagnosis when an LA mass is detected. Prompt surgical resection prevents thrombotic events and improves patient outcomes.

## Highlights

• Causes of left atrial thrombus include mitral valve disease and atrial fibrillation.
• Left atrial thrombi can be difficult to differentiate from left atrial tumors.
• Large left atrial thrombi can become free-floating or grow rapidly.
• For large left atrial thrombi, prompt surgery is required.

## 1 Introduction

LA thrombus, found in the LA appendage, is rarely found without the occurrence of atrial fibrillation (AF) and mitral valve disease (MVD) [1], [2]. When LA thrombi form in the left atrium and adhere to the atrial septum, they appear morphologically similar to LA tumors. We report a case where an LA ball thrombus was suspected to be an LA tumor in a patient with no history of AF or MVD. The patient underwent LA mass resection for a suspected LA tumor, which was actually a large thrombus, with a positive prognosis. The following case report is presented according to the standard guidelines [3].

## 2 Presentation of case

**Fig. 1:**
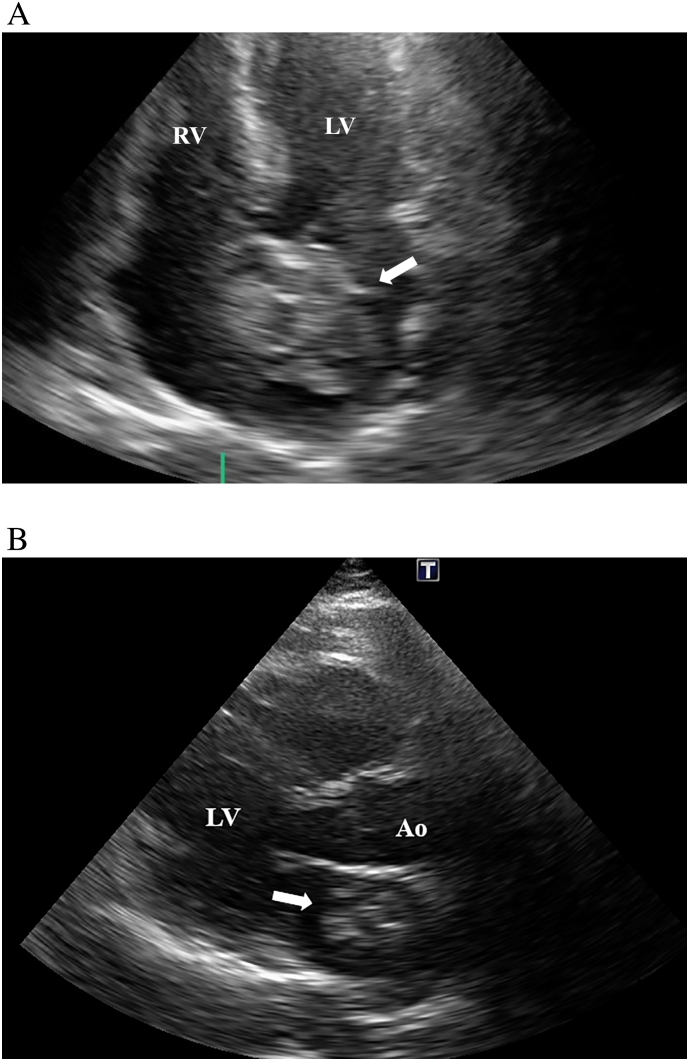
A. Transthoracic echocardiography imaging shows a large left atrial thrombus with calcification attached to the atrial septum (4 chamber view). B. Transthoracic echocardiography imaging shows a large left atrial thrombus with calcification attached to the atrial septum (long axis view).

**Fig. 2:**
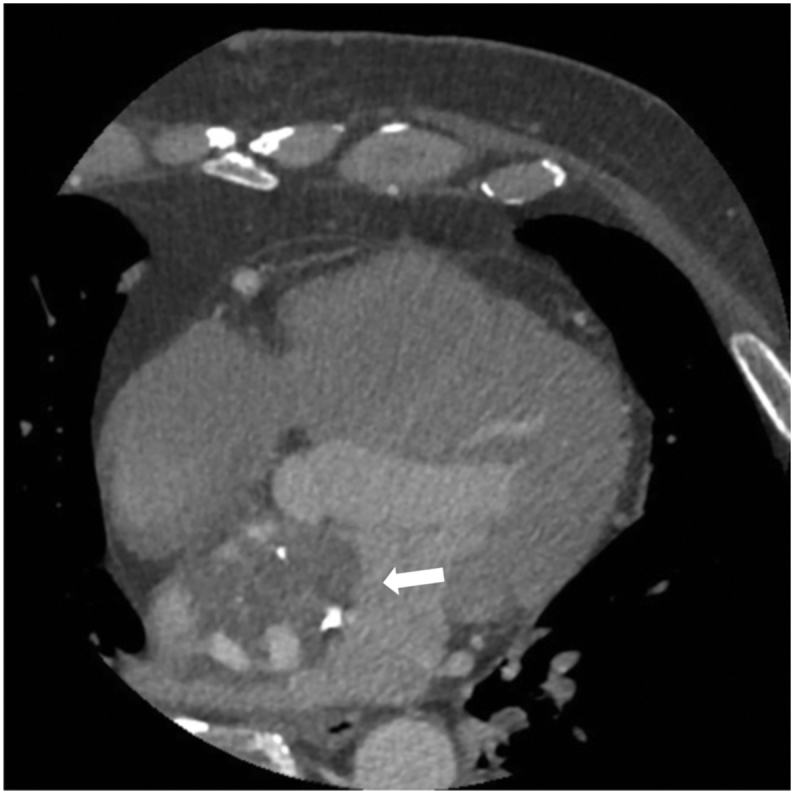
Computed tomography shows a large left atrial thrombus attached to the atrial septum.

A 63-year-old man with prior duodenal repair for duodenal ulcer and a mesh insertion for an abdominal incisional hernia was admitted with fever. He was otherwise asymptomatic. Additionally, he did not have any medical history of thrombotic events, like deep vein thrombosis, pulmonary embolism, transient ischemic attack, cardiovascular disease, or peripheral embolism. The patient had no history of smoking or drugs and no family history of thrombotic events. On physical examination, pus was observed in the patient's abdomen. Blood tests showed that the patient's calcitonin-related protein and fibrinogen levels were slightly elevated, at 2.63 mg/dL and 493 mg/dL, respectively. The anti-thrombin III level was within the normal range. Blood culture results were negative. The patient was diagnosed with a mesh infection, and 4-week antibiotic therapy was administered. He was transferred to our hospital for abdominal surgical debridement. Preoperative transthoracic echocardiography revealed a 39-mm mass in the LA adherent to the atrial septum (Fig. 1A, B), with an ejection fraction of 56% and no valvular abnormalities. Computed tomography also showed a 39-mm mass in his left atrium with partial calcification (Fig. 2). Brain magnetic resonance imaging revealed minuscule, scattered cerebral infarctions. Therefore, we suspected that the LA mass was a myxoma, and tumor resection was promptly scheduled.

**Fig. 3:**
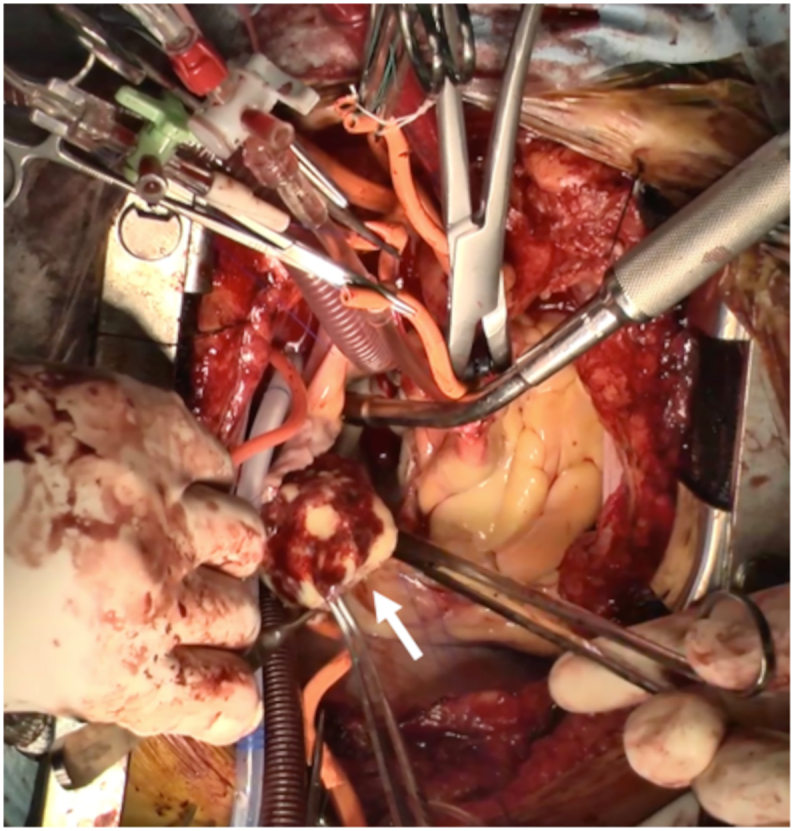
Intraoperative image of the large left atrial thrombus, adherent to the left atrium and subsequently surgically excised.

**Fig. 4:**
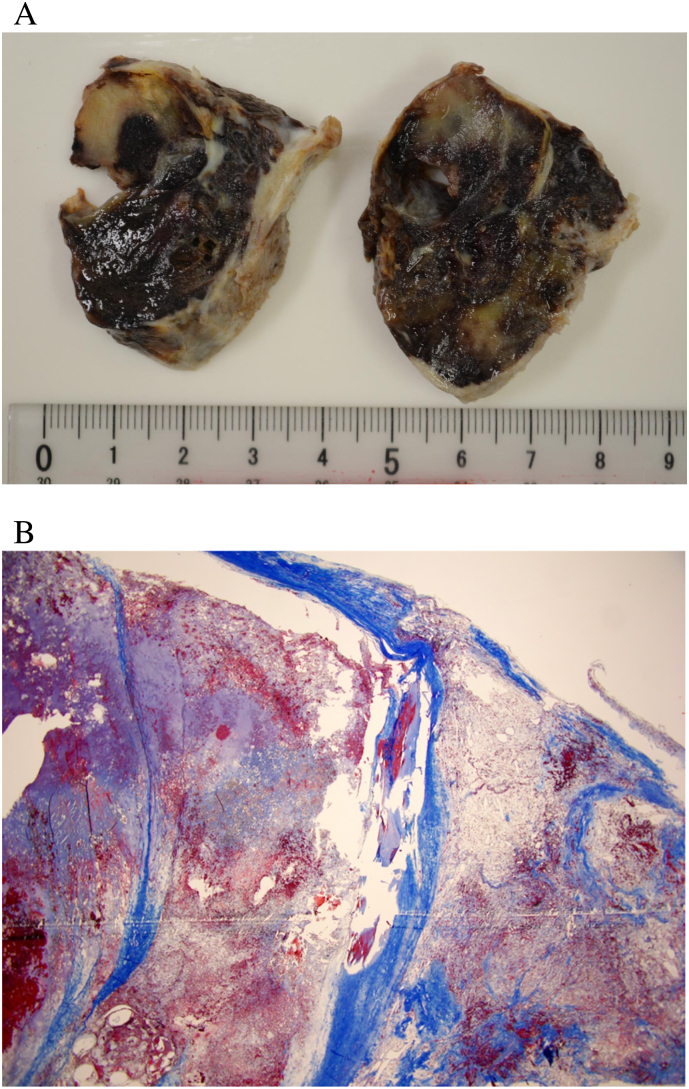
A. Resected left atrial thrombus composed of layered structures with Zahn lines. B. Pathology specimen showed the thrombus components and Zahn line composed of fibrin. No tumor pathology was observed in the thrombus.

The operation was performed by a senior resident (Dr. HN) of our cardiovascular surgery team. A smooth-surfaced mass was observed in the left atrium, firmly adherent to the atrial septum, therefore requiring meticulous resection (Fig. 3). The pathophysiology revealed that the excised specimen was a thrombus consisting of a fibrin-composed layered structure with Zahn lines (Fig. 4A, B). No tumor pathology was observed. The patient was administered direct oral anticoagulants to prevent thrombus formation and discharged on postoperative day 14. One year post-surgery, a follow-up transthoracic echocardiography showed no residual mass in the left atrium and a preserved ejection fraction of 55%. The patient was convinced that the treatment plan followed for the patient effectively prevented stroke and sudden death. He had no symptoms after surgery.

## 3 Discussion

LA thrombus, a common cause of cerebral infarction, is caused by blood stasis in the left atrium. The most common causes are AF and mitral valve stenosis. However, 6% of the patients with LA thrombus are in sinus rhythm, and 11% of the patients are not diagnosed with concomitant MVD [1], [2]. There are other causes of LA thrombus associated with blood flow congestion in the left atrium, including congestive heart failure lacking atrial kick, a large left atrium, and bradycardia [4].

The cause of the LA thrombus in this case was not clear. The echocardiography showed normal ejection fraction, normal size of the left atrium and left ventricle, and no signs of heart failure, thereby eliminating congestive heart failure. Patent foramen ovale also causes LA thrombus, but in such cases, the thrombus extends from the right atrium through the patent foramen ovale into the left atrium. This is not consistent with the present case [5]. Congenital coagulopathy is a potential cause but unlikely because of absent medical history and no family history of coagulopathy. Since oral anticoagulants were administered after surgery, detailed blood tests for coagulation could not be done. Paroxysmal AF could be another potential cause, but the absence of tachycardia and no detection of paroxysmal AF on Holter ECG eliminated this.

Cardiac tumors in the left atrium are mostly benign, frequently diagnosed as myxomas, lipomas, or papillary fibroelastomas. LA thrombi and LA tumors are nearly indistinguishable on echocardiography. Myxomas appear as smooth-surfaced and attached to the atrial septum by the stalk in the fossa ovalis. They present calcification-associated heterogeneous areas of hyper-echogenicity [6]. Similarly, in this case, the LA thrombus and LA tumor were indistinguishable on the echocardiogram. Contrast echocardiography can be used to differentiate between thrombi and myxomas effectively. Absent enhancement is indicative of thrombus, while partial enhancement suggests myxoma [7].

There are two notable characteristics of LA thrombus that differ from LA tumors. First, the incidence of embolic events is relatively high, at 10.4% per year [8], and LA thrombus grows by repeated adhesion to and release from the LA wall. Thus, even if the LA thrombus is temporary adherent to the wall, it can become a free-floating ball thrombus, causing embolism and sudden death if it blocks the mitral valve [9]. Second, the LA thrombus can grow rapidly. Morisaki et al. reported a left intra-atrial thrombus that rapidly grew from 2.5 cm to a 5.5 cm three-layered thrombus in 48 h [10]. In this case, the chronic mesh infection potentially may increase the overall coagulability of the system, causing the rapid formation of an LA thrombus over one month, consequently requiring prompt surgical intervention.

Ogata et al. reported that anticoagulation therapy might be effective in patients with small thrombus, small LA diameter, and fresh thrombus [11], but is not recommended for large left heart masses, including large smooth surface LA thrombi, because of increased risk of embolization [12]. Tanoue et al. reported an unusual case of a free-floating LA thrombus that disappeared after 23 days of anticoagulation therapy without complications. However, the thrombus broke into two during the treatment, further indicating that the thrombus-associated risk is high. Thus, anticoagulation therapy should be used only in patients deemed high-risk for surgery [13]. The average time between the diagnosis of LA thrombus by echocardiography and the onset of embolism is 53 days [8]. Surgical resection should be performed promptly for large LA thrombi. If anticoagulation therapy is used, the thrombus size should be checked after two weeks. In case of no improvement, surgical intervention is essential.

## 4 Conclusion

The occurrence of LA thrombus is rare without the diagnosis of AF or MVD. However, LA thrombus must be included in the differential diagnosis when an LA mass is detected. Prompt surgical resection prevents thrombotic events and significantly improves patient prognoses.

## Sources of funding

This research did not receive any specific grant from funding agencies in the public, commercial, or not-for-profit sectors.

## Ethical approval

N/A.

## Consent

Written informed consent was obtained from the patient for the publication of this case report and accompanying images. A copy of the written consent is available for review by the Editor-in-Chief of this journal upon request.

## Author contribution

HN: cared for the patient and wrote the report. HN and HW: read and approved the final version of the report. YH: collected the patient data.

## Research registration

N/A.

## Guarantor

Hironobu Nishiori.

## Declaration of competing interest

None.
